# Reproductive outcomes after fertility preservation using tamoxifen or letrozole in women with breast cancer: a long-term follow-up

**DOI:** 10.1016/j.xfre.2026.01.006

**Published:** 2026-02-05

**Authors:** Bo F. Paans, Madelon van Wely, Catharina C.M. Beerendonk, Jan Peter de Bruin, Astrid E.P. Cantineau, Taghride Dahhan, Nicole F. Klijn, Leonie A. Louwe, Jesper M.J. Smeenk, Mariëtte Goddijn, Eva M.E. Balkenende

**Affiliations:** a Center for Reproductive Medicine, Amsterdam University Medical Center, Amsterdam Reproduction and Development, Amsterdam, the Netherlands; b Department of Obstetrics and Gynaecology, Radboud University Medical Center, Nijmegen, the Netherlands; c Department of Obstetrics and Gynaecology, Jeroen Bosch Hospital, Den Bosch, the Netherlands; d University of Groningen, University Medical Center Groningen, Groningen, the Netherlands; e Department of Reproductive Medicine, University Medical Center Utrecht, Utrecht, the Netherlands; f Department of Gynaecology, Leiden University Medical Centre, Leiden, the Netherlands; g Department of Obstetrics and Gynaecology, St Elisabeth Hospital, Tilburg, the Netherlands

**Keywords:** Breast carcinoma, ovarian stimulation, pregnancy, tamoxifen, letrozole

## Abstract

**Objective:**

To evaluate long-term pregnancy outcomes in young women with breast cancer after ovarian stimulation for fertility preservation, with the addition of tamoxifen or letrozole to standard stimulation vs. no addition.

**Design:**

Follow-up study of the STIM-RCT, which was a multicentre randomized open-label trial (NTR4108), and the STIM-cohort study.

**Subjects:**

Women, aged 18–43 years, previously diagnosed with breast cancer who opted for fertility preservation through ovarian stimulation. All surviving women were eligible for follow-up (N = 216) and received an online questionnaire; 141 women from the STIM-RCT and 75 from the STIM-cohort.

**Exposure:**

Women receiving ovarian stimulation with the addition of tamoxifen, letrozole, or no addition.

**Main outcome measures:**

Natural conception, conception through assisted reproductive technology (ART), pregnancy rates, and live birth rates.

**Results:**

Out of 95 STIM-RCT responders, 36 (38%) received the addition of tamoxifen, 32 (34%) received letrozole, and 27 (28%) no addition (mean follow-up 7 years). Forty-four women became pregnant at least once after cancer treatment, and overall 7 women (7%) used their cryopreserved oocytes or embryos. In total, 63 pregnancies followed, of which 48 (76%) were conceived naturally, i.e., unassisted. Compared with standard ovarian stimulation, adding tamoxifen or letrozole resulted in comparable pregnancy rates in women who had at least one pregnancy after cancer treatment (tamoxifen vs. standard relative risk [RR], 0.92; 95% confidence interval [CI], 0.54–1.58; letrozole vs. standard RR, 0.84; 95% CI, 0.48–1.50), and live birth rates thereafter (tamoxifen vs. standard RR, 0.81; 95% CI, 0.54–1.22; letrozole vs. standard RR, 0.82; 95% CI, 0.53–1.26). In the STIM-cohort, 20 women became pregnant at least once, leading to a total of 30 pregnancies, of which 90% were conceived naturally. One woman used her cryopreserved embryos.

**Conclusion:**

After 7 years of follow-up, most women conceived naturally after cancer treatment, and 8 women used their cryopreserved oocytes or embryos. We found no evidence for a difference in pregnancy rates when adding tamoxifen or letrozole to standard ovarian stimulation compared with no addition.

Breast cancer is the most commonly diagnosed cancer in women worldwide, accounting for 32% of all cancer cases (1). Among women aged 30–39 years, it remains the most frequently diagnosed malignancy, and it is the leading cause of cancer death in young women (2). Advancements in cancer therapy have significantly improved survival rates. As a result, fertility preservation, using ovarian stimulation to bank oocytes or embryos, has become an increasingly standard practice in young women with breast cancer over the past decade (3, 4, 5, 6, 7, 8). Several (inter)national guidelines advise adding tamoxifen or letrozole to standard ovarian stimulation in women with breast cancer, to neutralize estrogen exposure and diminish the risk of tumor growth (9, 10, 11, 12, 13). To date, there is no evidence suggesting an increase in breast cancer mortality or recurrence, but international consensus on whether tamoxifen or letrozole should be routinely added to standard stimulation is lacking (14, 15, 16, 17).

Follow-up data on reproductive outcomes in women with a history of breast cancer who banked their oocytes or embryos, and whether they conceived naturally, i.e., unassisted, or conceived by assisted reproductive technology (ART), have only emerged in the past decade (7, 18, 19, 20). The potential impact of the different ovarian stimulation protocols on reproductive and pregnancy outcomes remains unclear. Several studies investigated the outcome of fertility preservation in terms of oocyte yield when adding tamoxifen or letrozole compared with standard ovarian stimulation, and found no differences in the number of oocytes retrieved (9, 12, 21). Regarding reproductive outcomes, most studies restricted their focus only on one specific ovarian stimulation protocol or only reported pregnancy outcomes regarding ART after cancer treatment (10, 13, 19, 20, 22). Long-term follow-up studies addressing reproductive and pregnancy outcomes in women with a history of breast cancer who opted for fertility preservation using ovarian stimulation with either addition of tamoxifen or letrozole, are currently lacking. It is important to provide clear evidence regarding reproductive and pregnancy outcomes for these women to help them make informed decisions about whether to pursue fertility preservation or not.

Therefore, this study aimed to provide evidence on reproductive and pregnancy outcomes in breast cancer survivors who previously cryopreserved their oocytes or embryos before receiving cancer treatment. Furthermore, this study will specifically examine the effects on pregnancy and live birth rates when tamoxifen or letrozole is added to standard ovarian stimulation protocols compared with no addition.

## Materials and methods

### Study design

This is a follow-up study, using a questionnaire, of the previously published STIM-RCT and STIM-cohort. The STIM-RCT was conducted in eight participating centers in the Netherlands and one center in Belgium, and investigated the effectiveness of ovarian stimulation with the addition of tamoxifen or letrozole compared with standard ovarian stimulation without the addition of tamoxifen or letrozole in women with breast cancer undergoing fertility preservation (9, 23). The STIM-cohort included women undergoing fertility preservation who declined participation in the STIM-RCT, however, granted their data to be used for research purposes. In the STIM-cohort, women were subjected to ovarian stimulation according to the local protocol of their hospital. This included either receiving standard ovarian stimulation with the addition of tamoxifen or letrozole, or standard ovarian stimulation without addition of tamoxifen or letrozole. For this reason, there was a possibility that type and dosage of medication differed among the participating centers, which is distinct from the participants in the STIM-RCT (23).

### Study population

Initially, all women included in the STIM-RCT and STIM-cohort were eligible for follow-up. This encompassed 241 women in total (9). However, because of the termination of the STIM-study in Belgium at UZ hospital Brussels after completion of the STIM-RCT, we were unable to send the questionnaire to 8 women from the STIM-RCT and one woman from the STIM-cohort of this hospital. In total, 16 women were deceased. Therefore, the surviving population that could be reached with a questionnaire consisted of 216 women. One patient of the STIM-cohort recently initiated palliative care and was therefore lost to follow-up after receiving the questionnaire (Supplemental Fig. 1, available online).

### Inclusion and exclusion criteria

Women were previously included when aged between 18 and 43 years, diagnosed with confirmed breast cancer, regardless of estrogen receptor status, and opted for fertility preservation through ovarian stimulation (23). The included women had given informed consent to be approached for future follow-up studies. If women did not want to participate in the follow-up study, they could either indicate so at time of signing the informed consent forms, or when they received the questionnaire. Women who did not give consent for follow-up were excluded. Before sending out the questionnaire, we extracted data from the Dutch Personal Records Database to verify participants’ addresses and confirm their life status, ensuring that women who were deceased would not receive the questionnaire.

### Study procedures

Participants were approached via an online questionnaire using the program ‟Open Source Software LimeSurvey” between January 2023 and September 2024. We opted for a digital questionnaire to pursue a high response rate. Patients who were included for follow-up received the questionnaire either via email with a link to the survey, or a written letter with a QR code to facilitate filling out the survey. All women received a unique study code, which they had to insert before filling out the questionnaire. Several questions were addressed in the questionnaire, such as return of menstrual cycle, using contraceptives after ovarian stimulation, desire to conceive, conceiving naturally or using ART. Questions regarding pregnancy addressed the number of pregnancies, pregnancy complications, pregnancy outcomes, mode of delivery, delivery complications, and birth weight.

### Outcomes

Outcomes addressed regularity of menstruation cycle, use of contraceptives, natural conception or through ART (either by using cryopreserved oocytes/embryos, or a new treatment), pregnancy rates, live birth rates, term at delivery, birth weight, pregnancy or delivery complications, and future purposes of the cryopreserved oocytes or embryos. Outcomes regarding safety of ovarian stimulation with addition of either tamoxifen or letrozole will be addressed in another manuscript.

### Statistical analysis

Collected data were exported from LimeSurvey to IBM SPSS 28 for further analyses. Data from the STIM-RCT and STIM-cohort were described separately. Differences in baseline characteristics were calculated using analysis of variance or χ^2^. Pregnancy rates were calculated as a proportion of those who actively tried to conceive. Live birth rates were calculated as proportions of all women who had at least one pregnancy after cancer treatment, and as a proportion of the total number of pregnancies. For women who had at least one pregnancy after their cancer treatment, with live births thereafter, we calculated relative risks (RRs) with 95% confidence intervals (CIs) to compare the addition of tamoxifen or letrozole with no addition during ovarian stimulation.

### Ethical approval

The Medical Research Ethics Committee confirmed that the Medical Research Involving Human Subjects Act (WMO) does not apply to this study (18.144 12-04-2018). Therefore, an official approval of the study was not required. All procedures used in this study comply with the principles of the Declaration of Helsinki.

## Results

In total, 216 women received the questionnaire. Of these women, 31 (14%) declined participation and 129 (60%) filled out the questionnaire. No response was received from the other 56 (26%) women. In the STIM-RCT, 141 women received the questionnaire, and 95 (67%) participated. In the STIM-cohort, 34 out of 75 women (45%) filled out the questionnaire (Supplemental Fig. 1).

### Participant characteristics

Baseline characteristics for the STIM-RCT and STIM-cohort are shown in Table 1. Of the 95 responders in the STIM-RCT, 36 (38%) received the addition of tamoxifen, 32 (34%) received the addition of letrozole, and 27 (28%) women received no addition to standard stimulation. All baseline characteristics were comparable between the different stimulation groups (Table 1). In the STIM-cohort all women received standard stimulation according to the local hospital protocol.

**Table 1:** Baseline characteristics of women in the STIM-RCT and STIM-cohort follow-up.

Baseline characteristics	STIM-RCT N = 95	Tamoxifen N = 36	Letrozole N = 32	Standard N = 27	*P* value	STIM-Cohort N = 34
Mean age at randomization, years	32 ± 4	31 ± 4	33 ± 4	31 ± 4	.35	32 ± 4
Current mean age, y	38 ± 4	37 ± 4	39 ± 4	38 ± 5	.35	39 ± 4
Parity					.29	
0	66	28	21	17		19
≥1	24	6	11	7		0
Missing	5	2	0	3		15
Follow-up time, y	7 ± 2	7 ± 2	7 ± 2	7 ± 1	.84	7 ± 1
Relationship status					.64	
Single	13 (14)	6 (17)	4 (13)	3 (11)		4 (12)
Male partner	78 (84)	30 (83)	25 (78)	24 (89)		27 (79)
Female partner	1 (1)	0	1 (3)	0		1 (3)
Missing	1 (1)	0	2 (6)	0		2 (6)

*Note:* Data are reported as mean ± SD or absolute numbers (%). SD = standard deviation.

### Reproductive outcomes

**Figure 1:**
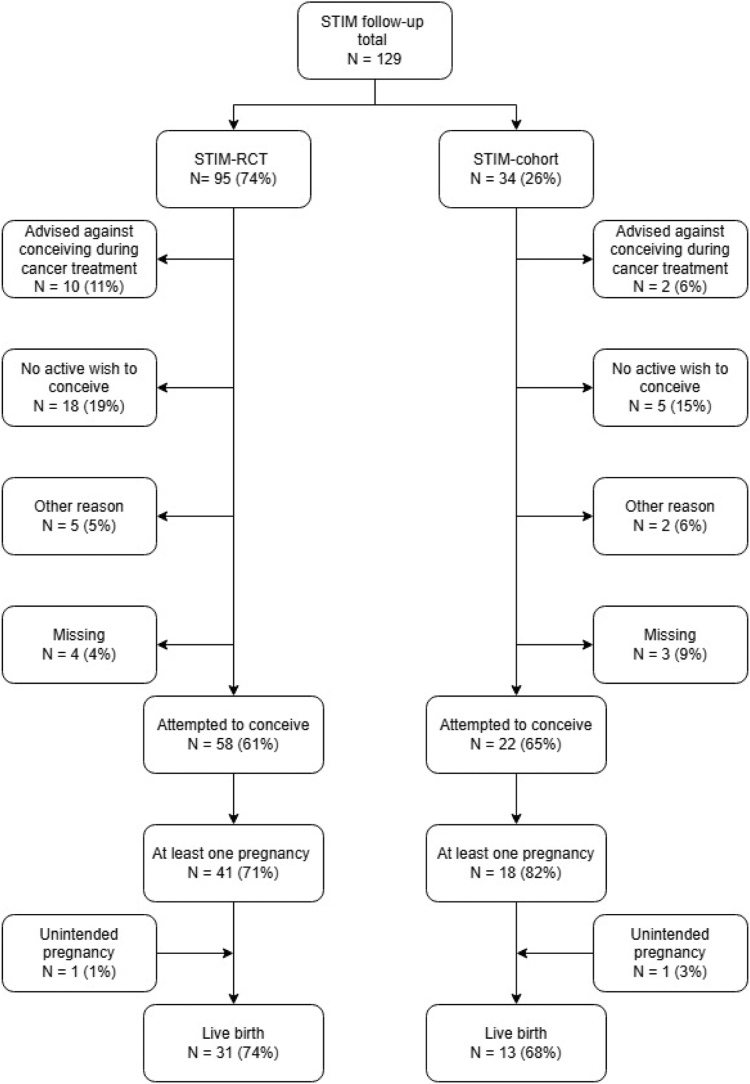
Flowchart of women who attempted pregnancy and the number of women who had their first pregnancy after breast cancer treatment, with live birth rates.

After cryopreservation of oocytes or embryos and completing or pausing endocrine therapy to pursue pregnancy, 58 women (61%) in the STIM-RCT and 22 women (65%) in the STIM-cohort indicated they actively tried to conceive. Of the women who attempted to conceive in the STIM-RCT, 41 (71%) had at least one pregnancy after cancer treatment. In the STIM-cohort, 18 women (82%) had at least one pregnancy after cancer treatment. In the STIM-RCT as well as the STIM-cohort, one woman reported an unintended pregnancy. This resulted in a total of 42 women having at least one pregnancy after cancer treatment in the STIM-RCT, which resulted in 31 (74%) live births. In the STIM-cohort, 19 women had at least one pregnancy after cancer treatment, which resulted in 13 (68%) live births (Fig. 1). According to the questionnaire, 47 (49%) women in the STIM-RCT indicated they had a regular menstrual cycle. In the STIM-cohort, 21 (62%) women indicated they had a regular menstrual cycle. Most women indicated they did not use contraceptives in both the STIM-RCT (N = 61, 64%) as well as the STIM-cohort (N = 17, 50%) (Table 2).

**Table 2:** Primary and secondary outcomes subdivided for the STIM-RCT and STIM-cohort follow-up.

Primary and secondary outcomes	Total N = 129	STIM-RCT N = 95	STIM-cohort N = 34
Menstruation cycle			
Regular cycle, n (%)	68 (53)	47 (49)	21 (62)
Irregular cycle, n (%)	26 (20)	20 (21)	6 (18)
Missing, n (%)	35 (27)	28 (30)	7 (21)
Contraceptive use			
No contraception, n (%)	78 (60)	61 (64)	17 (50)
Contraceptive pill, n (%)	5 (4)	4 (4)	1 (3)
Mirena/Kyleena IUD, n (%)	4 (3)	3 (3)	2 (6)
Copper IUD, n (%)	22 (17)	16 (17)	6 (18)
Condoms, n (%)	14 (11)	9 (9)	6 (18)
Missing, n (%)	4 (3)	2 (2)	2 (6)
Pregnancy attempt after cryopreservation			
Yes, n (%)	80 (62)	58 (61)	22 (65)
No, n (%)	43 (33)	33 (35)	10 (29)
Missing, n (%)	6 (5)	4 (4)	2 (6)
No. of pregnancies after cryopreservation			
≥1, n (% of women who attempted to conceive)	64 (80)	44 (76)	20 (91)
No. of pregnancies after cancer treatment			
First pregnancy, n	61	42	19
Second pregnancy, n	22	15	7
Third pregnancy, n	6	4	2
Fourth pregnancy, n	4	2	2
Mode of conception			
Total no. of pregnancies, n	93	63	30
Missing, n (%)	2 (2)	2 (3)	0
Natural, n (%)	75 (81)	48 (76)	27 (90)
ART, n (%) via:	16 (17)	13 (21)	3 (10)
Embryo transfer with cryopreserved embryos, n (%)	3 (19)	2 (15)	1 (33)
ICSI with cryopreserved oocytes, n (%)	5 (31)	5 (38)	0
IUI, n (%)	6 (38)	5 (38)	1 (33)
IVF, n (%)	2 (13)	1 (8)	1 (33)

*Note:* Women did not indicate whether they used cryopreserved embryos for transfer or fresh embryos.

ART = assisted reproductive technologies; ICSI = intracytoplasmic sperm injection; IUI = intrauterine insemination; IUD = intrauterine device; IVF= in vitro fertilization.

### Pregnancy outcomes

Twenty-two women reported several pregnancies (Table 2). This resulted in a total of 63 pregnancies in the STIM-RCT. Of these 63 pregnancies, the majority, 48 (76%) were conceived naturally vs. 13 (21%) who were conceived after using ART (Table 2). Of the 13 pregnancies conceived using ART, 2 (15%) were conceived using cryopreserved embryos and 5 (38%) using cryopreserved oocytes. Whereas 6 (46%) were conceived using intrauterine insemination (IUI) or in vitro fertilization (IVF) (Table 2). Of these 6 pregnancies, it was not specified whether the women still had their cryopreserved oocytes or embryos stored. Resulting from all pregnancies, 44 infants were born, and the overall live birth rate was 70% (Table 3). Mean gestational age of the newborns in the STIM-RCT at birth was 40 ± 1 weeks, and mean birth weight 3,664 ± 362 g.

**Table 3:** Pregnancies and pregnancy outcomes for the STIM-RCT per treatment group and STIM-cohort follow-up.

Pregnancy outcomes	STIM-RCT N = 95	Standard + tamoxifen N = 36	Standard + letrozole N = 32	Standard stimulation N = 27	STIM-Cohort N = 34
At least one pregnancy after cancer treatment, n	42	16	13	13	19
Live births, n (%)	31 (74)	11 (69)	9 (69)	11 (84)	13 (68)
Miscarriage, n (%)	5 (12)	2 (13)	2 (15)	1 (8)	5 (26)
Abortion, n (%)	2 (5)	1 (6)	–	1 (8)	1 (5)
Ectopic pregnancy, n (%)	1 (2)	–	1 (8)	–	–
Currently pregnant, n (%)	3 (7)	2 (13)	1 (8)	–	–
Total no. of pregnancies^a^, n	63	20	24	19	30
Live births, n (%)	44 (70)	14 (70)	16 (67)	14 (74)	18 (60)
Miscarriage, n (%)	10 (16)	2 (10)	5 (21)	3 (16)	8 (27)
Abortion, n (%)	2 (3)	1 (5)	–	1 (5)	2 (7)
Ectopic pregnancy, n (%)	2 (3)	–	2 (8)	–	1 (3)
Currently pregnant, n (%)	5 (8)	3 (15)	1 (4)	1 (5)	1 (3)

a Sum of all pregnancies women reported after cryopreserving their oocytes or embryos.

In the STIM-cohort, a total of 30 pregnancies were conceived, of which 27 pregnancies (90%) were conceived by natural conception (Table 2). Three pregnancies (10%) were conceived using ART. One pregnancy was conceived using IUI, and one pregnancy was after IVF treatment with genetic testing because of BRCA1 mutation. It was not specified whether these women still had their cryopreserved oocytes or embryos stored. One woman conceived after embryo transfer with oocyte donation after using all the cryopreserved embryos. Of all pregnancies in the STIM-cohort, 18 infants were born and the live birth rate was 60% (Table 3). Mean gestational age of the newborns in the STIM-cohort at birth was 40 ± 1 weeks and mean birth weight 3,671 ± 618 g. Reported complications during pregnancy or labor can be found in Supplemental Table 1 (available online).

### Pregnancy outcomes per stimulation protocol

Women who participated in the STIM-RCT received standard ovarian stimulation with the addition of either tamoxifen or letrozole, or no addition. Table 3 shows pregnancy numbers with pregnancy outcomes and live birth rates for each treatment group, separately presented per intention-to-treat. Compared with standard stimulation alone, the addition of tamoxifen or letrozole did not decrease the chances of having at least one pregnancy after breast cancer treatment (tamoxifen vs. standard: RR, 0.92; 95% CI, 0.54–1.58 and letrozole vs. standard: RR, 0.84; 95% CI, 0.48–1.50). Similarly, live birth rates after these pregnancies were not decreased in the tamoxifen and letrozole groups, respectively (RR, 0.81; 95% CI, 0.54–1.22 and RR, 0.82; 95% CI, 0.53–1.26) when compared with standard stimulation alone (Supplemental Table 2, available online).

### Future purposes of banked oocytes/embryos

Finally, all women were asked about future purposes of their frozen oocytes/embryos and whether women wished to donate them to science or other infertile couples if they would not use them. Approximately 1 in 4 women indicated they wish to use their frozen oocytes or embryos if they are unable to conceive naturally, and 12% indicated they already used all their frozen material. For future purposes, 20% wish to donate their frozen oocytes or embryos to science, and 14% to other infertile couples. These results are shown in Supplemental Table 3 (available online).

## Discussion

In the current long-term follow-up study, with a mean follow-up of 7 ± 2 years, we found that of women who attempted to conceive, approximately 80% got pregnant at least once after cancer treatment. Overall live birth rate was 67% with ongoing pregnancies at the time of completing the questionnaire. Furthermore, we found no evidence for a difference in pregnancy or live birth chances when adding tamoxifen or letrozole to standard stimulation.

### Strengths and limitations

To our knowledge, this is the first study reporting on reproductive and pregnancy outcomes comparing the addition of tamoxifen and letrozole to no addition in women with a history of breast cancer. Our results, with sufficient follow-up time, are promising for young women facing breast cancer who wish to start a family after cancer treatment. Especially, because most of these women (97% in the STIM-RCT and 73% in the STIM-cohort) received gonadotoxic treatment with risk of amenorrhea (6, 24, 25) (Supplemental Table 4, available online). However, because we did not use a standard case report form, a lot of data regarding breast cancer treatment are missing. Another strength is that we systematically approached these women using a questionnaire, which provided information on whether women had a desire to get pregnant, and whether women conceived naturally or used ART after cancer treatment. In this way, our study might be more informative on the actual pregnancy rate and outcomes, because many women who experience early miscarriage for example, never seek a caregiver and these pregnancies are usually not registered in medical files.

Our study also had limitations that should be considered when interpreting the results. First, although our study had a fixed number of women who could be approached after the initial STIM-trial and STIM-cohort were completed, no power calculation was made beforehand. Therefore, our study has limited power to prove a small difference because of the low response rate. Second, because our study relies on a questionnaire, recall bias may have occurred. Third, our findings showed a high chance of natural conception, which might be because of the younger age of women (mean age 32 ± 4 years) when they were diagnosed with breast cancer in our cohort. As previously described, women with breast cancer who are aged >40 years are prone to have a return of their menstrual cycle after cancer treatment (6). On the contrary, we found a relatively high miscarriage percentage of 19%, which could be attributed to women being older when they tried to conceive compared with the general population (26). Unfortunately, we did not have exact data at what age these women got pregnant. Also, there was limited data available on time to become pregnant after or when pausing breast cancer treatment, and the use of banked oocytes or embryos, because not all women filled out the entire questionnaire. Finally, women received different breast cancer treatments, such as different chemotherapy regimens or endocrine therapy, which makes it challenging to draw conclusions on the return of ovarian function for the entire breast cancer population.

### Pregnancy outcomes

Expressed per woman who tried to conceive, we found that 76% of women in the STIM-RCT, and 91% of women in the STIM-cohort got pregnant at least once. These findings are in line with the POSITIVE-trial, which studied women with hormone receptor-positive breast cancer who temporarily discontinued adjuvant endocrine therapy to become pregnant, and found that 70.5% had at least one pregnancy (22). These data suggest that most women with breast cancer, who have permission to conceive after their cancer treatment or during a temporary interruption, are healthy enough to conceive which could be attributed to what is called the “healthy mother effect” (19). However, our study did not address whether women temporarily discontinued endocrine therapy or had oncological permission to conceive. Most pregnancies occurred after natural conception in our study, vs. conception by ART. Half of pregnancies conceived by ART were achieved by using cryopreserved oocytes or embryos. Of the other half, only one woman indicated she used all her cryopreserved embryos, whereas the other women who used ART did not specify whether they still had cryopreserved oocytes or embryos stored. Although numbers were low, more cryopreserved oocytes were used compared with cryopreserved embryos, most likely since after 2012 cryopreservation of oocytes became a standard practice compared with cryopreserving embryos (8).

When addressing live birth rates, our findings suggest a much higher live birth rate of 67% in total compared with other studies reporting on pregnancy outcomes after breast cancer treatment in general, ranging from 20%–27% (18, 27, 28, 29). An explanation might be that our follow-up time was up to 9 years. A Swedish study found a cumulative incidence of live births in breast cancer survivors of 19.4% after 5 years, which increased to 40.7% at 10 years (29). However, this study only reported on live birth rates after ART, whereas our study also reported on natural conception. This could explain the higher live birth rate in our population. Furthermore, over the years, breast cancer treatments advanced, and some women in our population had permission to take a break from their adjuvant treatment to become pregnant, which could have resulted in more live births because more women had permission to conceive before they faced the end of their reproductive potential.

### Pregnancy outcomes per stimulation protocol

Compared with standard stimulation alone, we found no differences between the addition of tamoxifen or letrozole in having at least one pregnancy and live birth after cancer treatment. It is difficult to conclude whether the different stimulation protocols have had an influence on pregnancy and live birth rates in this specific study. Biologically, it is unlikely that these additions would have had an influence because they are only administered for approximately 2 weeks during ovarian stimulation and would then only have an effect on the cryopreserved oocytes or embryos. Our study showed that most women conceived naturally, making the influence of these additions even more unlikely. Few studies have been published on pregnancy outcomes after ovarian stimulation using tamoxifen or letrozole. Dezellus et al. (13) performed a 5-year follow-up, which is now extended to 10 years in 95 women who received ovarian stimulation, all with the addition of tamoxifen. This study found a live birth rate of 22% after natural conception with a mean time interval of 2.8 years after diagnosis. Seven percent returned for embryo transfer with frozen-thawed oocytes (13). Although this study also took natural conception into account, it did not elaborate whether women had a desire to become pregnant at that time. This could explain the lower number of live births, because some women may not have had a desire for pregnancy 3 years after their diagnosis. Oktay et al. (10) performed a long-term follow-up of 14 years in women who received ovarian stimulation with the addition of letrozole and found an overall live birth rate of 45% per embryo transfer. They did not include women who conceived naturally. After these results, it is hardly possible to draw conclusions regarding the addition of tamoxifen or letrozole to standard ovarian stimulation, because so many different factors are involved in becoming pregnant and eventually delivering a healthy infant.

### Utilization of banked oocytes/embryos

A recent national study investigated the utilization rate of cryopreserved oocytes or embryos in the Netherlands, comparing categories of indication of fertility preservation (7). The study consisted of 1,112 women, of whom 693 had an oncological indication, with a follow-up of 10 years. Of the oncological indications, 69% had breast cancer. In this study, a quarter of women had returned to use their cryopreserved oocytes or embryos. It was not specified how many women with a history of breast cancer returned, yet the study demonstrated that clinical pregnancy rate and live birth rate were lowest for the oncologic indications (7). In our study, only 8 (6%) out of 129 women indicated they returned to use their cryopreserved material. Because this national study had a follow-up of 10 years and was conducted in 10 IVF clinics in the Netherlands, there is a potential for overlap with the women included in our study and probably a higher return rate because of the longer follow-up.

## Conclusion

Our findings show favorable pregnancy outcomes in breast cancer survivors after an average follow-up of 7 years. Out of all pregnancies in our cohort, approximately two-thirds resulted in live births, and some ongoing pregnancies during follow-up. Approximately 1 in 5 women required ART and half of these women used their cryopreserved oocytes or embryos. The other half used IUI or IVF and did not indicate whether they still had cryopreserved oocytes or embryos stored. These results aid in clinical counseling regarding high chances of natural conception while also informing women they might require the use of their cryopreserved oocytes or embryos in the future. However, it is very difficult to predict which women would need to use their cryopreserved oocytes or embryos. To provide breast cancer survivors with clear, evidence-based guidance on reproductive and pregnancy outcomes, more research is necessary to optimize stimulation protocols, improve counseling, and clinical decision-making. Future studies should address larger cohorts with longer follow-up, to investigate if women with a history of breast cancer not only conceive once, but can complete their desired family in the long run.

## Declaration of Interests

B.F.P. has nothing to disclose. M.v.W. is Editor-in-Chief of Human Reproduction Update. C.C.M.B. has nothing to disclose. J.P.d.B. has nothing to disclose. A.E.P.C. has nothing to disclose. T.D. has nothing to disclose. N.F.K. has nothing to disclose. L.A.L. has nothing to disclose. J.M.J.S. reports funding from unrestricted grant from Merck and Ferring outside the submited work; honorarium from Merck BV; and travel suppport from Merck BV and Goodlife BV. M.G. has nothing to disclose. E.M.E.B. has nothing to disclose.
