# Supplementary material for: Reproductive outcomes after fertility preservation using tamoxifen or letrozole in women with breast cancer: a long-term follow-up
Source: F S Rep. 2026 Feb 5;7(2):125–33. doi: 10.1016/j.xfre.2026.01.006 (PMC13100848; doi:10.1016/j.xfre.2026.01.006)
Supplement: Supplementary material [file mmc1.docx]

**Supplemental figure 1.** Flowchart: follow-up of the STIM-RCT and STIM-cohort.


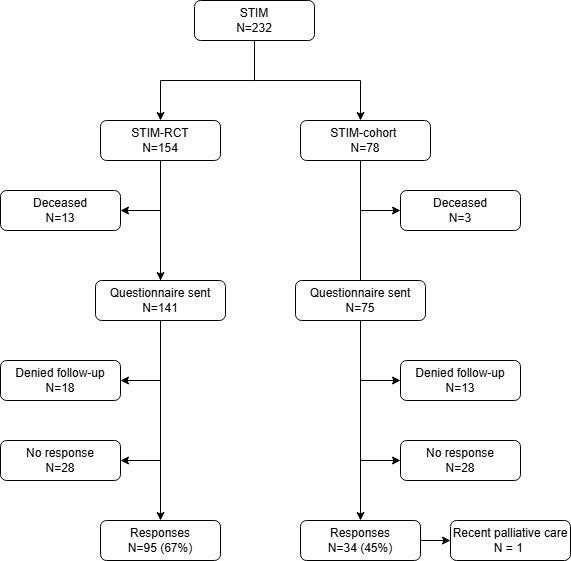


| **Supplemental table 1.** Reported complications during pregnancy or labour | | |
| --- | --- | --- |
|  | **STIM-RCT**  N=63 | **STIM-cohort**  N=30 |
| **Pregnancy induced diabetes, n(%)** | 4 (6) | 0 |
| **Pregnancy hypertension, n(%)** | 4 (6) | 0 |
| **Pre-eclampsia, n(%)** | 5 (8) | 1 (3) |
| **Growth restriction, n(%)** | 1 (2) | 1 (3) |
| **Infection during labour, n(%)** | 2 (3) | 0 |
| **C-section, n(%)** | 3 (5) | 0 |
| **Total, n(%)** | 19 (30) | 2 (7) |

| **Supplemental table 2.** Relative risks (RR) for women in the STIM-RCT having at least one pregnancy after cancer treatment and live birth thereafter. CI= confidence interval. | | |
| --- | --- | --- |
|  | **RR** | **95%CI** |
| **At least one pregnancy after cancer treatment**  Tamoxifen vs. standard  Letrozole vs. standard | 0.92  0.84 | 0.54-1.58  0.48-1.50 |
| **Live births following at least one pregnancy after cancer treatment**  Tamoxifen vs. standard  Letrozole vs. standard | 0.81  0.82 | 0.54-1.22  0.53-1.26 |

| **Supplemental table 3.** Usage and intention for future purposes of the cryopreserved oocytes/ embryos, subdivided for the STIM-RCT and STIM-cohort. | | | |
| --- | --- | --- | --- |
|  | **Total**  N=129 | **STIM-RCT**  N=95 | **STIM-cohort**  N=34 |
| **Use of frozen oocytes/embryo’s**  I was not able to freeze oocytes/ embryo’s, n(%) | 6 (5) | 5 (5) | 1 (3) |
| I will not use my frozen oocytes/ embryo’s, n (%) | 29 (22) | 26 (27) | 3 (9) |
| I will use my frozen oocytes/ embryo’s if I cannot conceive naturally, n(%) | 33 (26) | 24 (25) | 9 (26) |
| I have used all my frozen oocytes/ embryo’s, n(%)  Missing, n(%) | 16 (12)  45 (35) | 11 (12)  29 (31) | 5 (15)  16 (47) |
| **Future purpose**  Donation to science, n(%) | 26 (20) | 24 (25) | 2 (6) |
| Donation to other infertile couples, n(%) | 18 (14) | 13 (14) | 5 (15) |
| Destruction of frozen oocytes/ embryo’s, n(%) | 16 (12) | 11 (12) | 5 (15) |
| I don’t know, n%)  Missing, n(%) | 50 (39)  19 (15) | 33 (35)  14 (15) | 17 (50)  5 (15) |

| **Supplemental table 4.** Breast cancer treatment characteristics. TAC = Taxotere, Adriamycin, and Cyclophosphamide; AC = Adriamycin, Cyclophosphamide; FEC = Fluorouracil, Epirubicin hydrochloride, and Cyclophosphamide; GnRH = Gonadotropin-releasing hormone. | | |
| --- | --- | --- |
| **Breast cancer therapy characteristics** | **RCT** | **Cohort** |
|  | N = 108 | N = 37 |
| Finished treatment, n (%)  Missing, n(%) | 70 (65)  12 (11) | 27 (73)  2 (5) |
| Surgery, n(%)  Missing, n(%) | 101 (94)  4 (4) | 9 (24)  28 (76) |
| Lumpectomy, n(%)  Missing, n(%) | 48 (44)  16 (15) | 5 (14)  32 (87) |
| Mastectomy, n(%)  Missing, n(%) | 55 (51)  7 (7) | 3 (8)  34 (92) |
| Mastectomy contralateral breast, n(%)  Missing, n(%) | 16 (15)  11 (10) | -  37 (100) |
| Radiotherapy, n(%) | 67 (62) | 7 (19) |
| Unknown, n(%) | 6 (6) | - |
| Missing, n(%) | 5 (5) | 30 (81) |
| Chemotherapy timing,  Adjuvant, n(%)  Neoadjuvant, n(%) |  |  |
|  | 23 (21) | 1 (3) |
|  | 74 (69) | 10 (27) |
| Missing, n(%) | 11 (10) | 26 (70) |
| Chemotherapy type |  |  |
| TAC, n(%) | 14 (13) | 1 (3) |
| AC, n(%) | 53 (49) | - |
| FEC, n(%) | 7 (7) | - |
| Other, n(%) | 19 (18) | - |
| Missing, n(%) | 15 (14) | 36 (97) |
| Immunotherapy, n(%) | 24 (22) | 1 (3) |
| Missing, n(%) | 51 (47) | 36 (97) |
| Endocrine therapy |  |  |
| Tamoxifen, n(%) | 53 (49) | 10 (27) |
| Missing, n(%) | 43 (39) | 27 (73) |
| Letrozole, n(%) | 3 (3) | 2 (5) |
| Missing, n(%) | 58 (54) | 35 (95) |
| GnRH agonist, n(%) | 25 (23) | - |
| Missing, n(%) | 59 (55) | 37 (100) |
